# Improving healthcare quality by unifying the American electronic medical report system: time for change

**DOI:** 10.1186/s43044-024-00463-9

**Published:** 2024-03-15

**Authors:** Roopa Kumari, Subhash Chander

**Affiliations:** Department of Pathology, Icahn School of Medicine at Mount Sinai, 1 Gustave L. Levy PI, New York, NY 10029 USA

## Abstract

**Background:**

In recent years, innovation in healthcare technology has significantly improved the efficiency of the healthcare system. Advancements have led to better patient care and more cost-effective services. The electronic medical record (EMR) system, in particular, has enhanced interoperability and collaboration across healthcare departments by facilitating the exchange and utilization of patient data. The COVID-19 pandemic further accelerated this trend, leading to a surge in telemedicine services, which rely on electronic communication to deliver healthcare remotely.

**Main body:**

Integrating artificial intelligence (AI) and machine learning (ML) in healthcare have been instrumental in analyzing vast data sets, allowing for identifying patterns and trends that can improve care delivery and pinpoint potential issues. The proposal of a unified EMR system in the USA aims to capitalize on these technological advancements. Such a system would streamline the sharing of patient information among healthcare providers, improve the quality and efficiency of care, and minimize the likelihood of errors in patient treatment.

**Conclusion:**

A unified electronic medical record system represents a promising avenue for enhancing interoperability within the US healthcare sector. By creating a more connected and accessible network of patient information, it sets the stage for a transformation in healthcare delivery. This change is imperative for maintaining the momentum of progress in healthcare technology and realizing the full potential of recent advancements in patient care and system efficiency.

## Background

The modern healthcare system has witnessed a remarkable change by transforming service delivery mechanisms using patient-centered, value-based, and coordinated approaches [1]. As a result of this evolution, a tremendous shift is expected in the next decade in the approach and delivery of the healthcare system fostered by an immense rise in healthcare costs, growth in consumerism, and digital transformation. Most importantly, a critical contribution to the changing dynamics of the healthcare system has been made by electronic medical records (EMR). Although there is no consensus definition of EMR, the World Health Organization defines EMR as “a real-time patient health record with access to evidence-based decision support tools that can be used to aid clinicians in decision-making” [2]. EMR may include patient information for clinical applications such as contact details, medical history, allergies, diagnostic test results, treatment plans, and non-clinical applications such as billing and disease surveillance [2, 3]. It differs from an electronic health record (EHR), a longitudinal record of patient data generated during one or more clinical encounters containing additional information such as clinician’s notes during each encounter [2].

In the USA (US), health information technologies such as EMR adoption have been incentivized by the American Recovery and Reinvestment Act of 2009 to reduce healthcare costs and medical errors by streamlining clinical workflow [4]. However, there has been considerable resistance to the widespread adoption of EMR in US hospitals due to concerns surrounding additional clerical tasks required for patient information documentation, poor usability of EMR systems, and physician burnout [5, 6]. Nonetheless, US hospitals adopting EMR have experienced small but significant improvements in the duration of hospitalization and 30-day mortality, albeit with an increase in 30-day rehospitalization in the two years after EMR adoption compared to hospitals that did not adopt EMR [4]. In addition, EMR has been shown to improve the quality of clinical information documentation, coordination, and safety [7]. It has significantly improved the quality of care, as evidenced by the provision of population health management tools and data analytics, enabling healthcare providers to identify recent trends and revolutions [8]. Thus, EMR has fostered disruptive innovation in the American healthcare system, with the technology expected to improve the functioning of healthcare organizations by 6% each year [9].

The recent COVID-19 pandemic highlighted several limitations of the existing EMR systems, perhaps the most important ones being the lack of automated notification to infection preventionists of suspected or confirmed COVID-19 and the non-centralized EMR with non-standardized user interface, which increased test volume and staff exposure while hindering patient tracking across health systems [10]. Hence, several researchers have underscored the need to urgently standardize and centralize EMR systems [10–12]. Therefore, this review aims to highlight the opportunities to enhance the implementation of a unified EMR for improved efficiency and leverage its potential to transform the American healthcare system.

## Main text

### Innovation and its relation to optimal healthcare provision

Innovation development in healthcare has paved paths toward improved system efficiency, quality of patient care, collaboration and communication mechanisms, and cost-effective healthcare services, increasing the overall efficiency of the healthcare system significantly [13].

### Recent trends—a perspective of EMR

According to the Office of the National Coordinator for Health Information Technology, as of 2021, over 90% of hospitals and 50% of clinical physicians have adopted and implemented some form of EMR system in their healthcare practice [14]. This trend has been driven by various factors such as government incentives, not-for-profit organizations, and the potential for improved care delivery and cost savings. In addition, the rise in consumerism in healthcare in terms of enhanced use of technology by individuals to manage their health allows them to track their health metrics and communicate with their healthcare providers [15]. This shows that EMR has a dominant positive role in healthcare transformation by increasing the affordability and accessibility of healthcare services while also leading toward improved efficiency and quality of patient care effectively.

### Effective usage practices and requirements

The COVID-19 pandemic led to the production of a massive volume of health data, igniting interest in the use of big data analytic tools and artificial intelligence (AI) to improve organizational issues in the healthcare system, predictive and prescriptive analytics, pandemic management, diagnosis, drug discovery, and treatment [16–18]. The ever-increasing use of AI and machine learning in healthcare has contributed effectively toward a rigorous and informed analysis of large amounts of data and identifying patterns or trends that may be useful for improving care delivery or identifying potential issues. For instance, AI algorithms can effectively increase the efficacy of prediction related to the likelihood of a patient developing a particular condition based on their medical history and other factors [19]. In addition, the use of other health information technologies, such as telemedicine, increased from 0.3% of all clinical encounters before the pandemic to 23.6% of all encounters in 2020 [20]. Given the timesaving and convenience of telemedicine coupled with the experience of physicians and patients in using telemedicine during the pandemic, the widespread use of such technologies is likely to continue in the post-pandemic era [21].

The leveraging of these technological advances relies significantly on interoperability and collaboration in healthcare departments by enabling different systems to exchange and use patient data. Hence, in addition to developing a centralized EMR with a standardized user interface, these recent trends in the use of health information technologies also require robust integration with EMRs.

### Healthcare dynamics—a regulatory perspective

Governments significantly influence healthcare through policy development, funding allocation, and shaping delivery models, which can both facilitate and hinder healthcare innovation [22]. For instance, the US Affordable Care Act of 2010 enhanced healthcare services access, improved care quality, reduced costs, and improved patient outcomes [23]. Notably, the US government played a crucial role in promoting EMR implementation, making it mandatory for hospitals to transition to digital format. It further invested $27 million as part of the Health Information Technology for Economic and Clinical Health (HITECH) Act [24], leading to a near-universal EMR implementation in US hospitals and demonstrating the government’s immense influence on healthcare innovation.

### Challenges to health innovation

Despite the numerous benefits, their growing need in the post-pandemic era, and the governmental push for widespread adoption, EMR’s effectiveness can be hindered by lack of unification, particularly in the US healthcare system [25]. For example, with the growing number of patients with multimorbidity, there has been a growing call to restructure the US primary care system to multidisciplinary care [26, 27]. Multidisciplinary care can include (i) collaboration through shared consultations, (ii) co-located teams of highly coordinated healthcare professionals but without shared consultations, (iii) collaboration via referral and counter-referral, which usually has a clinical leader who collates medical information from other specialists and guides the overall care of the patients, and (iv) non-hierarchical continuous horizontal collaboration which lacks a distinctive clinical leader [26]. While EMRs can significantly improve healthcare procedures, diagnoses, and symptom management in all four types of multidisciplinary care models, the use of proprietary formats and strict privacy protocols by different EMR systems can impede information sharing between healthcare organizations [28], especially when some of the team members are outside of the primary point of care [27, 29].

In addition, many patients may travel across state lines to avail medical facilities unavailable locally. For instance, about 8% of patients travel across state lines in the US to avail opioid treatment programs [30] or access abortion facilities [31]. The lack of non-centralized EMR means that the health data cannot be readily shared between healthcare practitioners beyond what is recalled or volunteered by the patient traveling across state lines to avail healthcare, which may exacerbate the quality of care and patient satisfaction.

Hence, it is essential to standardize data formats and develop interoperability national standards to maximize EMR benefits, enhance data quality, and boost healthcare accessibility and quality.

## Framework for EMR priority assessment

Priority assessment stands as a cornerstone in patient care, enabling healthcare professionals to access, analyze, and leverage information effectively. This process is pivotal in developing strategic plans that ensure patient stability and enhance the quality of care. The “Framework for EMR Priority Assessment” is introduced to systematize the approach utilizing EMRs to prioritize patient needs efficient and accurately. The structured framework assists healthcare providers in deciphering vast amounts of patients data, facilitating informed decision-making that leads to improve patient outcomes and healthcare delivery.

### Principles and priorities of the healthcare system

EMRs have been transformative in the American healthcare landscape, fostering a culture of creativity and innovation that significantly enhances healthcare quality and patient care. The mandate from the American Recovery and Reinvestment Act (2014) for healthcare institutions to implement EMRs and demonstrate their meaningful use marks a pivotal advancement. This includes enhancements in quality and safety, patient engagement, care coordination, and data security [32]. Since this mandate, the adoption of EMR has been prioritized for their superiority in facilitating access to information, reducing errors, and supporting decision-making processes. Incentive programs like meaningful use further underscore the US government’s commitment to EMT adoption, rewarding providers that leverage EMRs to elevate care quality [33]. Such initiatives underscore the shift toward a more insightful, data-driven approach in healthcare, aimed at improving patient outcomes while sustainably reducing cost.

### Challenges to EMR standardization and advanced implementation

While EMRs presents significant advantages, the journey toward their standardization and advanced implementation in the American healthcare system faces notable challenges [28]. Variabilities in data types, collection methods, and the unique functionalities of different EMR systems complicate the standardization process, often leading to interoperability issues within healthcare networks. The financial, hardware, and software demands of EMR standardization pose additional barriers, particularly for smaller or economically disadvantaged organizations. Concerns around data privacy and security further complicate the landscape, highlighting the need for a concerted effort among stakeholders to achieve a secure, efficient EMR ecosystem [33].

### Pathways to advanced EMR implementation—prospects and constraints

**Fig. 1:**
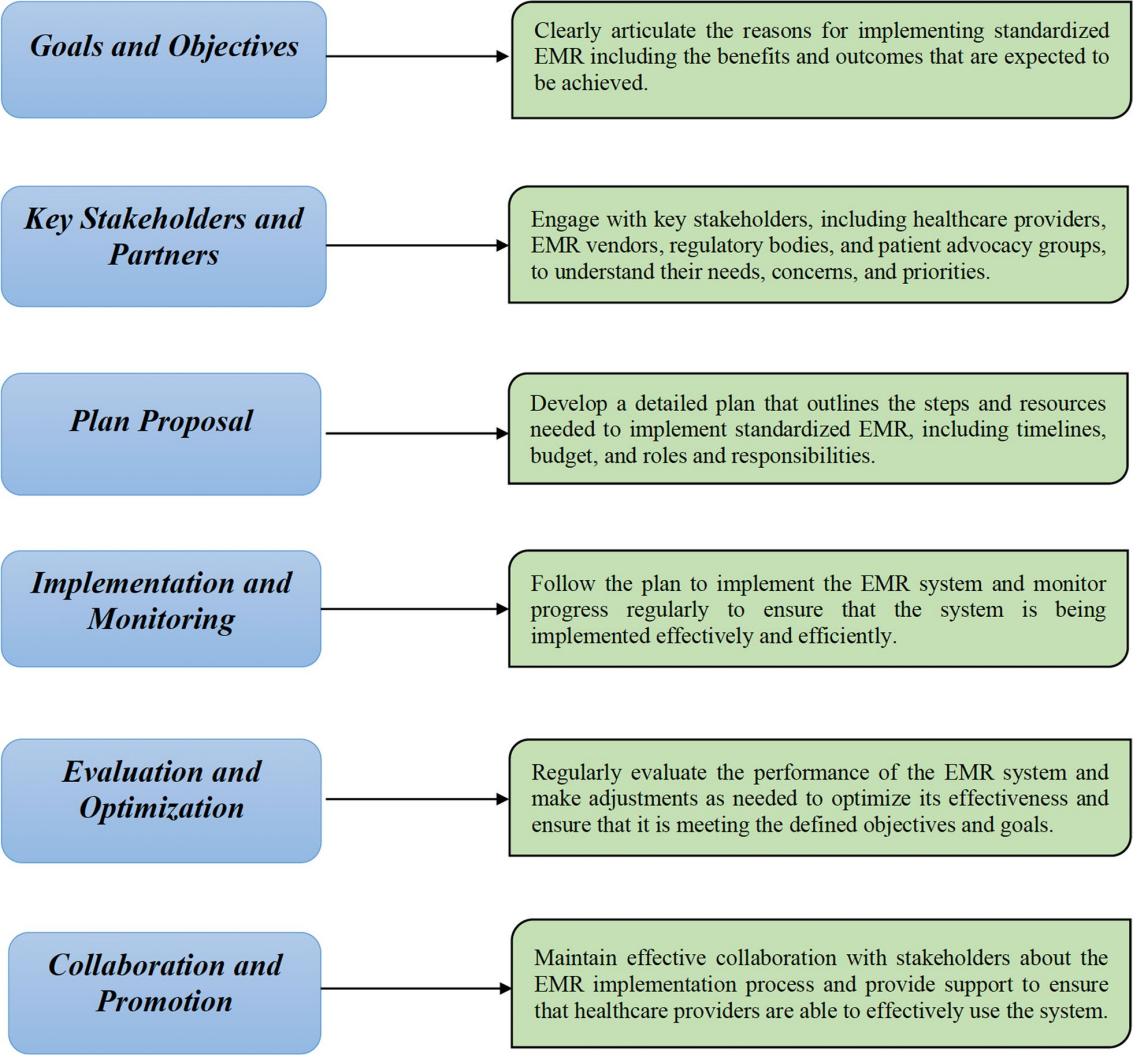
Pathway for advanced implementation of EMR

The ultimate goal of advanced EMR implementation in the USA is to streamline the way healthcare providers interact with patient information. By transitioning to a standardized EMR system, healthcare professionals can more swiftly access and share critical patient data, allowing them to focus on delivering care rather than managing paperwork or manual data entries. This shift not only aids in prioritizing healthcare activities, but also minimizes the risk of medical errors, contributing significantly to patient safety [34]. For this purpose, the following implementation pathway is suggested in Fig. 1.

### Incorporating real-life examples into the framework

To illustrate the proactive application and benefits of the “Framework for EMR Priority Assessment,” consider the following real-life scenarios:

1. *Improving Chronic Cardiac Condition Management:* A primary care clinic utilized the framework to identify patients with malfunctioning Intra Cardiac Devices (ICDs) by analyzing EMR data for early warning sings. This proactive approach enabled early intervention, significantly improving cardiovascular management and outcomes.
2. *Enhancing Emergency Care:* An emergency department adopted a framework designed to prioritize the discharge of borderline sick patients within 24 h, utilizing real-time data analysis to monitor pending laboratory results. This innovation streamlined the triage process, effectively reducing wait times and enhancing both patient satisfaction and outcomes.

These examples underscore the framework’s value in enhancing patient care by leveraging EMR data to make informed, prioritized decisions. By addressing the challenges of EMR standardization and embracing the potential of advanced implementations, healthcare providers can significantly improve the quality and efficiency of care delivery.

**Fig. 2:**
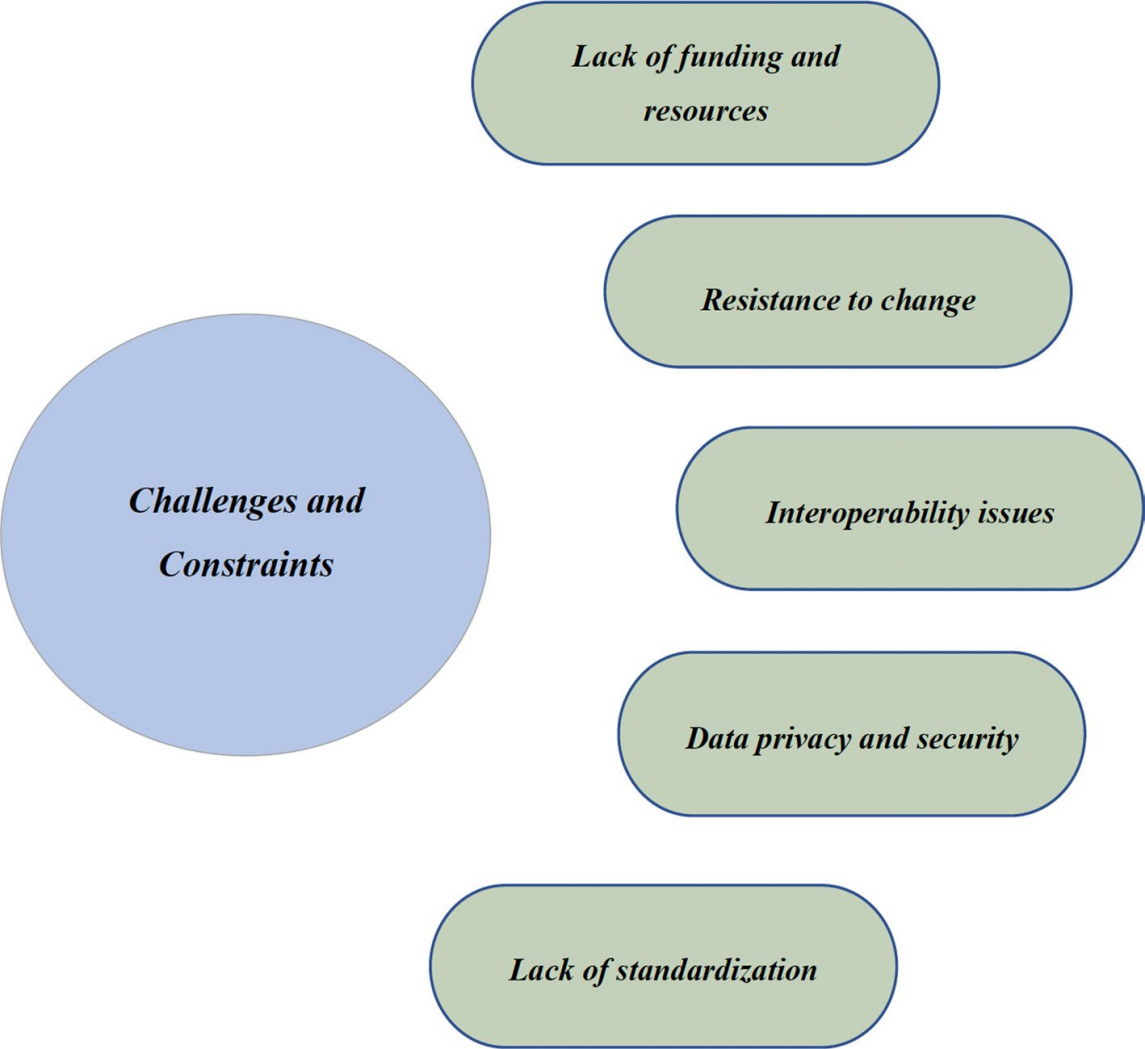
Challenges and constraints of EMR

A “Unified EMR System” envisioned in the strategic proposal described in Fig. 1 can generate competitive opportunities, enhance interoperability in US healthcare departments, and improve the quality and efficiency of care by making it easier for healthcare providers to access and share patient information as well as reducing the risk of errors and discrepancies, thereby supporting informed and better decision-making [35]. However, it is important to consider the challenges and constraints that can detract from the effectiveness of implementation and the associated positive outcomes (Fig. 2).

## Healthcare outcomes—advanced EMR perspective

Healthcare providers must strategize the effective implementation of an advanced EMR system as a lack of clear and concise planning can lead to the selection of a system that might not meet the needs of the relevant healthcare organization and its patients, hence resulting in a dominant decrease in user satisfaction, increased healthcare costs, and reduced efficiency [35]. Given this aspect, a comprehensive account of positive and negative outcomes associated with implementing a unified EMR system in the American Healthcare System can be developed.

### Positive outcomes

The positive outcomes associated with the implementation of advanced EMR are listed below.

*Improved Patient Care*—Unified EMR can provide healthcare providers access to a wide range of patient information and data, enabling them to make more informed decisions and efficiently foster quality care. It can help streamline healthcare processes, reduce the risk of errors and duplications, decrease the incidence of medical negligence, and improve the overall efficiency and effectiveness of healthcare delivery [36].

*Reduced Healthcare Costs*—Although EMR systems can be costly to implement and maintain, a cost–benefit analysis of EMR implementation in the primary care setting indicates that the payback of system implementation cost will occur by the end of the first year with a net positive return of USD 79,375–124,725 over five years [37]. Unified EMR can help reduce healthcare costs by automating routine tasks, reducing the need for manual record-keeping, enabling real-time data collection and analysis, reducing staff errors, and ensuring continuity of care during physician handoffs that can contribute toward the prioritization of healthcare activities and diversion of investment toward essential healthcare areas [37, 38]. Thus, the cost savings could be in the form of reduced transcription costs, chart pull costs, accurate reimbursement coding, reduced liability claims for organizations, reduced lost income, and lower healthcare costs for patients [37]. Estimates from nearly two decades ago indicate that the overall cost saving of EMR implementation and networking for just one organization (Georgia Lung Association) could be as high as USD 657,500 annually [39]. However, others estimate savings of up to USD 81 billion annually with more widespread adoption [40]. Consistent with these studies, a systematic review showed that, on average, the annual benefits of the EMR system amounted to 308.6% of its annual cost [41]. Although the cost–benefit analysis of a unified EMR has not been conducted, it is very likely to yield far more significant economic benefits in the post-pandemic era than previously estimated.

*Improved Patient Satisfaction-* Implementing a unified EMR will significantly eliminate the need to carry out repetitive patient assessments, diagnostic tests, and other examinations, contributing to enhanced patient safety and satisfaction [39]. This aspect will help patients become increasingly content with their care when they can access their medical records and track their health progress.

### Negative outcomes

The negative outcomes associated with the implementation of advanced EMR are listed below.

*High upfront costs*: An earlier study estimates that the EMR system implementation at a single practice can cost USD 213,083 [39]. Hence, implementing advanced unified EMR can be cost-intensive, specifically regarding the learning and development needs necessary to equip and train the healthcare team with the accurate and efficient usability of standardized EMR.

*Data privacy and security concerns*—Ensuring the security and privacy of patient data can be a significant challenge [42]. As EMRs store large volumes of sensitive patient information, they can be vulnerable to cyberattacks or unauthorized access, requiring the healthcare system to devise and implement methods for careful and informed access to information stored in EMRs.

## Suggested interventions to effective EMR implementation

A comprehensive implementation of a systematic framework consisting of three main elements concerning planning, execution, and monitoring will be required to counter the aforementioned challenges, constraints, and negative outcomes, which can significantly detract from the effective implementation and performance of standardized EMR in the US healthcare system.

### Standards of design and development

The identification and engagement with key stakeholders, including medical staff, IT staff, and administrative experts, will be required to gather input and ensure that the new system meets the needs and preferences of modern healthcare dynamics and addresses any concerns detracting from the execution of a unified EMR [11]. This will be followed by developing a clear and concise set of standards for the design and development of the EMR system concerning specifications for data storage, security, and interoperability with other systems. For this purpose, these standards will be based on industry best practices and align with relevant regulatory requirements such as the Health Insurance Portability and Accountability Act (HIPAA) [43].

### Monitoring and evaluation post EMR deployment

It is crucial to monitor and evaluate the performance of a standardized EMR system after deployment in the US healthcare system with its impact on patient care and clinical outcomes. While traditional metrics such as usage rates and user satisfaction are essential, they do not necessarily provide a complete picture of the value of the EMR system. By collecting data on the impact of the EMR system on patient outcomes, organizations can get a more comprehensive view of the system’s value. This might include gathering data on the frequency and severity of medical errors, patient satisfaction with care, and the time required to complete various tasks [43]. Hence, by regularly reviewing this data, organizations can identify areas where the EMR system positively impacts patient care and make any necessary adjustments to ensure that the system continues supporting high-quality care.

## Conclusion

This review provides an in-depth analysis and evaluation of positive and negative outcomes related to implementing unified EMR in the USA. With a unified EMR system, healthcare departments and medical staff can easily access and update patient records, reducing healthcare costs along with the time and effort required to manage patient information. This can lead to improved patient care, increased collaboration, and reduced medical errors achieved through enhanced interoperability of healthcare systems, making it easier for healthcare organizations to exchange information when patients receive care from multiple providers. In a nutshell, unified EMR will significantly contribute to reducing barriers to quality care and improving the quality and coordination of care for patients.

## Data Availability

Yes, on reasonable request.

## Abbreviations

AI: Artificial intelligence
EMR: Electronic medical record
HIPPAA: Health insurance portability and accountability act
HITECH: Health information technology for economic and clinical health
ML: Machine learning
